# National scale up of PROM based monitoring after joint replacement in Germany

**DOI:** 10.1038/s44401-026-00122-y

**Published:** 2026-07-08

**Authors:** Laura Wittich, Lukas Schöner, David Ehlig, Reinhard Busse, Justus Vogel

**Affiliations:** 1 Faculty of Economics and Management, Department of Health Care Management, Technische Universität Berlin, Berlin, Germany; 2 School of Medicine, Chair of Health Economics, Policy and Management, University of St. Gallen, St. Gallen, Switzerland

**Keywords:** Diseases, Health care, Mathematics and computing, Medical research

## Abstract

Patient-reported outcome measures (PROMs) can extend follow-up after joint replacement beyond discharge. Using Germany as a case example, national scalability and its potential impact on cost and health outcomes of a trial-proven PROM-based digital monitoring intervention were assessed. A four-step extrapolation mapped trial tasks and time, removed study-only procedures to derive streamlined delivery models, projected national implementation costs (personnel, licence fees, integration), and modelled population-level effects using trial outcomes and insurance claims. Personnel time decreased from 62 min per patient (trial) to 21 min in a hybrid model that retained limited human touchpoints and to 10 min in an automated model that restricted human involvement to safety-critical interventions, with costs of €158, €133 and €127, respectively. Assuming constant effectiveness, estimated health gains were 0.023–0.025 QALYs per patient with €218–€249 savings, suggesting potential annual national savings of €75–€86 million and a surplus of more than 8200 QALYs. Realised value depends on licence pricing, scalable integration, minimal human touchpoints for engagement and safety, and the transferability of trial effects to streamlined delivery models.

## Introduction

Across health systems, digital health interventions (DHIs) are promoted to personalise care, extend monitoring beyond clinical settings, and enhance patient engagement through timely and structured feedback^1^. In orthopaedics, such innovations are exemplified by digital applications that support post-operative monitoring by capturing patient-reported symptoms, identifying deviations from expected recovery, and triggering timely clinical responses. Patient-reported outcome measures (PROMs) have become central to these efforts, systematically documenting patients’ health status and outcomes from their own perspective^2^.

One of the most established examples of PROM implementation is the National PROMs Programme in the United Kingdom, which has systematically collected PROMs data for hip and knee arthroplasty since 2009. A comparable effort exists in the Netherlands, where PROMs are routinely collected through national registries such as the Dutch Arthroplasty Register to support quality monitoring and hospital benchmarking. In both systems, patients typically complete standardised questionnaires before and after surgery, often from home using digital or paper-based formats. The collected data inform not only the assessment of clinical outcomes and care quality but also hospital benchmarking and commissioning decisions across the health system^3–5^.

Germany is now advancing similar efforts by formally integrating PROMs into its national arthroplasty registry, the Endoprothesenregister Deutschland (EPRD)^6^. As one of Europe’s largest joint replacement registries, currently encompassing over 700 hospitals^7^, the EPRD is being expanded to include a standardised, digital collection of patient-reported data on pain, joint function, and health-related quality of life (HRQoL) before and after hip or knee arthroplasty. Since mid-2025, all participating hospitals have been required to adopt the updated EPRD system, which supports PROM collection during and after hospitalisation; routine PROM collection is encouraged but not yet mandatory. This development represents a significant shift toward patient-centred quality monitoring, thereby extending the registry’s scope and aligning it with international standards for continuous outcome monitoring.

While the expansion signals commitment to patient-centred care, it raises major implementation questions. Will PROM data be actively used to inform clinical decisions, or primarily serve documentation? How can hospitals integrate additional tasks into already stretched workflows? This ambiguity reflects a deeper tension in digital health implementation—between innovation and integration, between formal requirements and clinical reality—that extends beyond PROMs alone. Addressing this gap is critical for ensuring that digital tools contribute to lasting improvements in care, rather than becoming additional administrative burdens.

The implementation challenges surrounding PROMs for joint replacement surgery reflect broader obstacles that have characterised the implementation of DHIs in Germany, particularly the tension between regulatory requirements for evidence generation and the operational realities of healthcare delivery. Germany has taken a leading role in regulating DHIs. The 2019 Digital Care Act established a fast-track pathway for the reimbursement of digital applications under statutory health insurance^8^. This approval process requires manufacturers to demonstrate clinical benefit, typically through randomised controlled trials (RCTs), to ensure that DHIs meet the rigorous standards of evidence-based care. Demonstrating benefit is a prerequisite for reimbursement, confirming that these technologies are at least equivalent to standard care^9^.

Despite this promising regulatory infrastructure, early implementation has encountered considerable challenges. Health authorities have reported difficulties in assessing the real-world impact, with current evidence often deemed insufficient, particularly regarding structural and procedural improvements in care (e.g. workflow integration, interoperability, prescribing routines or use of DHI data)^10^. High costs associated with DHI deployment have also prompted scrutiny of whether these investments are justified, given the uncertain benefits in day-to-day clinical settings^11^.

Limited initial adoption among patients and healthcare providers^12^ further raises concerns about the implementation of DHI in routine care. These concerns underlie a paradox^12^: providers hesitate to adopt new technologies without robust evidence, yet such evidence cannot be generated without widespread adoption of the technology. This dilemma highlights the need for structured, practical strategies to translate trial-based innovations into routine clinical settings and to assess how trial outcomes can be meaningfully extrapolated to broader patient populations and complex healthcare systems. However, bridging the gap between controlled trials and the practical feasibility of implementation in routine care presents further challenges, many of which only become apparent when digital interventions are scaled and embedded within complex real-world systems^13^.

These questions echo broader tensions in the digital health landscape: between evidence and implementation, between regulatory demands and clinical realities. To explore these tensions, this paper draws on findings from the *PROMoting Quality* trial, a multicentre RCT conducted in nine German hospitals. The trial evaluated a PROM-based digital monitoring tool designed to detect atypical recovery paths following hip or knee replacement surgery. All patients received standard care and completed PROMs at admission, discharge, and 12 months post-surgery. In the intervention arm, additional PROMs were completed, triggering alerts and follow-up actions in response to atypical recovery patterns^14^. The tool, with its early-detection and remote-monitoring functions, represents a basic form of DHI^15^.

The intervention improved multiple health outcomes in patients, including higher HRQoL, better joint-specific function, and a greater proportion achieving a minimal clinically important difference (MCID)^16^. Mean healthcare expenditure over 12 months was approximately €375 lower per patient,^17^, attributable to reduced post-discharge utilisation, namely fewer outpatient contacts and physiotherapy after hip replacement, and fewer prescriptions and medical aids after knee replacement^18^.

However, favourable outcomes and lower costs in a trial setting do not by themselves establish feasibility at scale. The intervention relied on dedicated personnel and digital infrastructure that are not routinely funded in standard care^19^, and implementation in routine practice is likely to be constrained by staff workload, workflow adaptation, and sustained patient engagement, particularly in remote formats^20^. The relevant question, therefore, extends beyond whether the intervention appears favourable in cost-effectiveness terms under trial conditions to whether it can be delivered in routine care with acceptable resource requirements and maintained effects. Accordingly, the present study is best understood as an exploratory implementation and scalability analysis that incorporates cost-effectiveness metrics as one component and baseline of a broader assessment, rather than as a definitive economic evaluation in the narrow sense.

Using the PROMoting Quality trial as a case example, it translates trial evidence into operational requirements for routine care. Building on Murray et al.^21^, the analysis is organised around four practice-oriented questions. It first examines which intervention components proved feasible in the trial, and how they translate into deliverable tasks in routine care. It then distinguishes study-specific activities from those required for basic delivery of PROM-based monitoring, and uses this distinction to specify streamlined care models. Next, it estimates the personnel inputs, software licence fees and implementation costs are required for national scale. Finally, it assesses the intervention’s population-level health outcome in terms of quality-adjusted life years (QALYs), and what are the expected costs necessary to achieve this outcome.

## Results

**Fig. 1:**
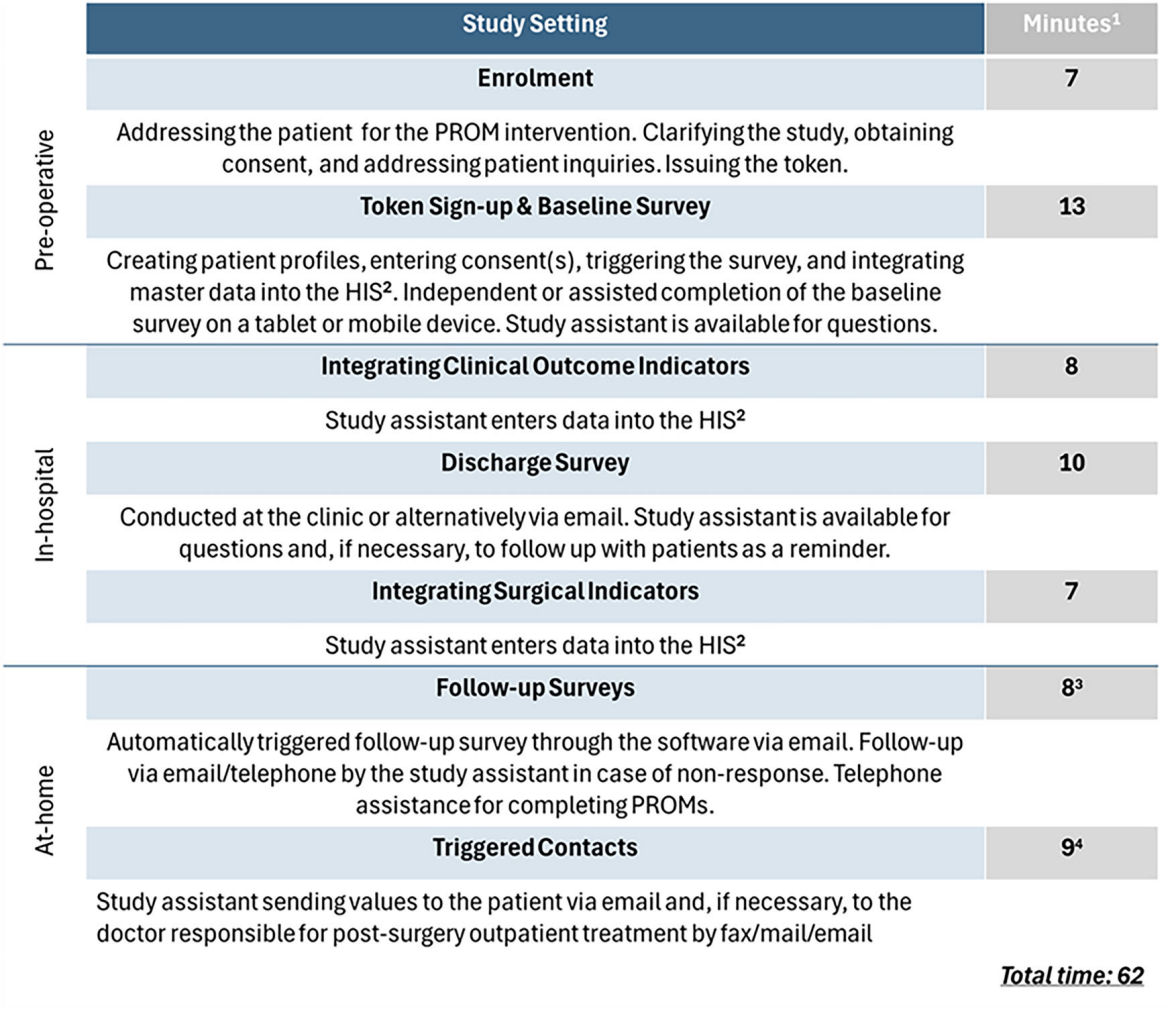
Intervention steps and associated time effort in the Trial Setting. ^1^Average minutes per task as indicated by the study assistants. ^2^HIS = Hospital Information System. ^3^Follow-ups were conducted at 1, 3, 6, and 12 months. In ~20% of cases, study personnel provided support (e.g. reminders or phone assistance), which required approximately 10 min per supported follow-up. Total support time: 10 × 0.2 × 4 = 8 min per patient. ^4^The intervention was applied at 1, 3, and 6 months (not at 12 months). On average, 24% of patients triggered a contact, each requiring ~12 min. Total intervention time: 12 × 0.24 = ~2.9 min per time point, multiplied by 3: 3 × 2.9 = ~8.7 min, rounded to 9 min per patient.

For Step 1, ‘Time and task analysis’, key components of the intervention were mapped and categorised across three phases of the care pathway: pre-operative, in-hospital, and post-discharge. Assistance encompassed seven main tasks distributed across these three phases. Each task carried out by study assistants during the trial was disaggregated and assigned an average time requirement based on structured survey responses from all participating hospitals and study assistants, as well as expertise input from the software provider (see Fig. 1).

During the **pre-operative phase**, assistants spent approximately 20 min per patient. Of this, 7 min were allocated to enrolment, which involved informing patients about the trial, addressing questions, and obtaining written consent for participation and for the use of their health insurance and hospital data, in accordance with ethical and data protection regulations. Another 13 min were spent on baseline setup and survey support. The latter included issuing a token for software access, creating a patient profile, triggering the baseline survey, and manually entering data into the hospital information system (HIS), which was particularly time-consuming in hospitals that could not automatically import patient data. Patients completed the survey independently on a tablet or their own device, with assistants available for support as needed.

In the **in-hospital phase**, assistants entered clinical outcome indicators (8 min), supported the discharge survey (10 min), and entered surgical data (7 min).

In the **post-discharge phase**, 8 min were allocated for follow-up survey support, including follow-up emails or phone reminders for non-responders, as well as assistance for specific groups (e.g. elderly patients). Where symptom thresholds were exceeded, 9 min were spent on triggered contacts, including communicating results to patients and, if necessary, forwarding summary sheets to follow-up care providers.

In total, delivering the intervention in the Trial Setting required 62 min of assistant time per patient over 12 months.

For Step 2, ‘Development of streamlined implementation models’, the feasibility of the intervention in routine care was assessed by reviewing assistant tasks and separating essential clinical functions from trial-specific procedures. Trial-only activities (e.g. written trial consent, pseudonymised token issuance, manual entry into the HIS) were excluded. On this basis, two streamlined implementation models were specified: a Hybrid Care model, which retains selective personal contact at onboarding and follow-up, and an Automated Care model, which shifts onboarding and follow-up to digital automation. In both models, limited personnel involvement remained for enrolment confirmation and clinical alert review to support participation and patient engagement.

- **Hybrid care model**: Purely trial-related tasks, such as enrolment or combining different administrative data sources, are eliminated in this scenario. It only preserves selected personal contact points, particularly during onboarding and follow-up, to support patient engagement. It assumes that a limited degree of human interaction helps sustain adoption and effectiveness, consistent with the original Trial Setting.
- **Automated care model**: This model maximises digital automation and minimises personnel requirements by transferring routine tasks to a digital assistant, comparable to a digital coach. Such a system would guide patients through the onboarding process, provide step-by-step support for completing PROMs, and send automated reminders via email or SMS. The digital coach replaces in-person follow-up, reducing reliance on personnel while maintaining user engagement through interactive, responsive prompts. Only the review and response to symptom-triggered alerts remain the responsibility of personnel, ensuring that critical deviations in recovery trajectories receive appropriate attention. This leaner setup preserves the feedback loop essential for clinical safety, while demonstrating how PROM-based monitoring could function within a predominantly digital care model.

**Fig. 2:**
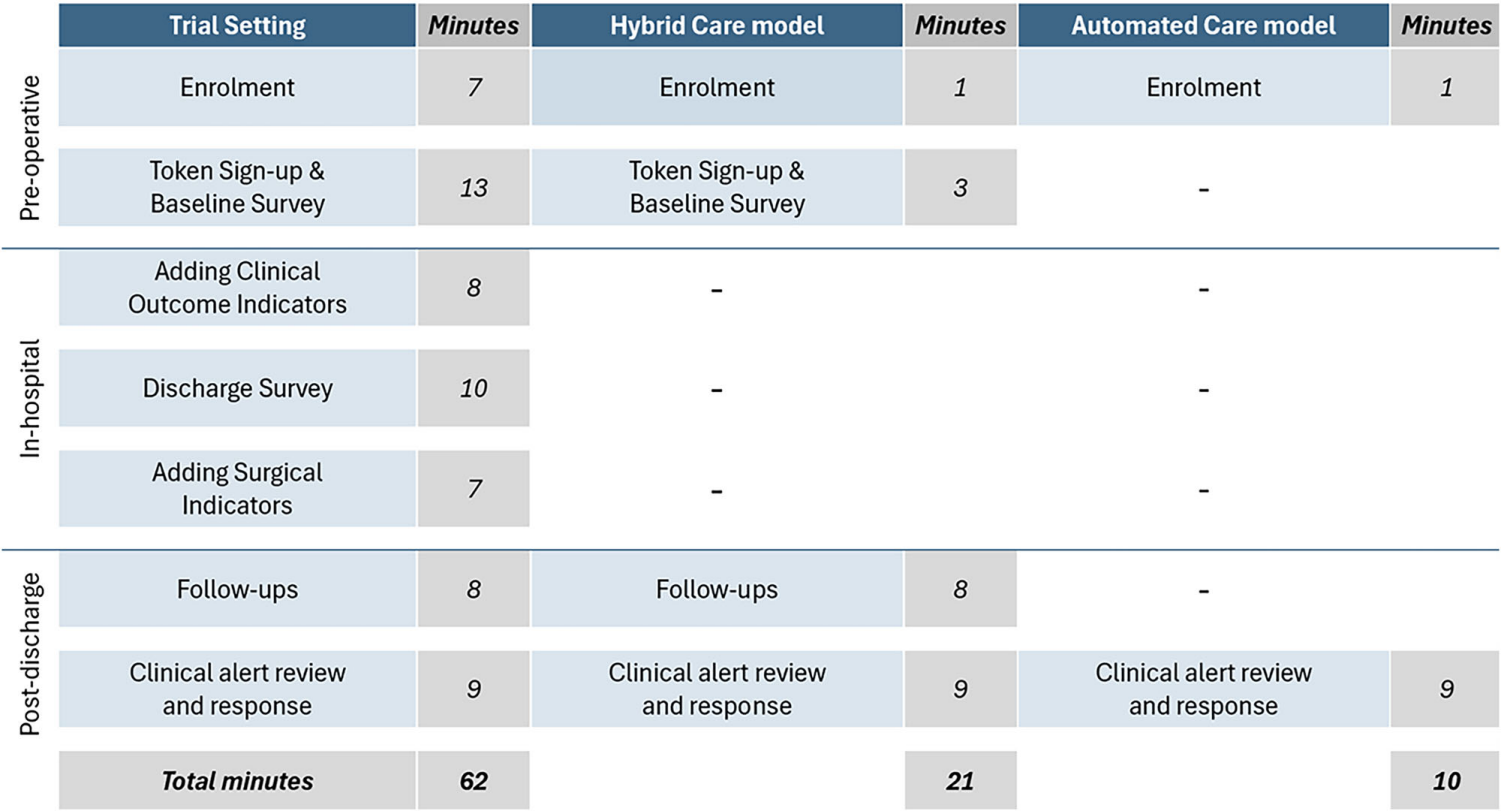
Comparison of intervention steps and associated time effort in the Trial Setting, Hybrid Care model, and Automated Care model. The figure contrasts tasks and per‑patient minutes across three phases. Pre‑operative, in‑hospital, and post‑discharge. Total minutes: Trial 62, Hybrid 21, Automated 10. Dashes indicate steps not performed in that model.

Both models retain minimal assistant involvement during enrolment and clinical alert response, as these touchpoints are considered critical for maintaining patient motivation and ensuring clinical safety. Figure 2 summarises these tasks and time reductions across the care pathway for all three models.

### Personnel time requirements were reduced as follows

- **Pre-operative phase**: In the Trial Setting, the pre-operative phase required approximately 20 min per patient: about 7 min for study enrolment and consent, and about 13 min for baseline set-up and survey support. In the streamlined implementation models, procedures specific to the research context, such as separate trial enrolment, detailed study documentation, manual data entry into the HIS, and issuance of pseudonymized access tokens, are omitted. These are replaced by processes compatible with routine care: consent for PROM-based electronic monitoring is embedded in the standard hospital admission workflow, and required patient identifiers are automatically pulled from existing administrative systems via interface integration, eliminating time-intensive manual data entry.
  Under the Hybrid Care model, the integrated consent reduces the consent step from approximately 7 min to about 1 min, and assistants provide brief technical support for baseline setup (about 3 min), yielding a total pre-operative assistant time of roughly 4 min.
  Under the Automated Care model, consent takes approximately 1 min, onboarding is completed independently within the app, and personnel input is limited to a brief explanation and device handover, resulting in a total time of about 1 min.
- **In-hospital phase**: All in-hospital tasks—entry of clinical data, discharge support, and documentation (25 min)—were trial-specific and removed in all streamlined models, as they did not contribute directly to PROM-based monitoring.
- **Post-discharge phase**: The Hybrid Care model retained personal contact during follow-up (8 min) and clinical alert review and response (9 min), totalling 17 min. In contrast, the Automated Care model automated all follow-up processes, maintaining only 9 min for alert review and response.

Overall, the personnel time required for the intervention was reduced from 62 min per patient in the trial to 21 min in the Hybrid Care model and 10 min in the Automated Care model.

For Step 3, ‘Scalability’, the assessment–beyond the structural adjustments described in the care models**–** involved projecting personnel and technology-related costs for each model. The cost projection included three components of the total per-patient estimate: personnel costs, software licensing fees, and one-time implementation costs for system setup and integration.

**Personnel costs** were calculated by applying the average time required per patient—62 min in the Trial Setting, 21 min in the Hybrid Care model, and 10 min in the Automated Care model—to the standard wage rate for nursing personnel in Germany (€0.59 per minute).

**Licensing fees** were estimated at €100 per patient, based on information provided by the software provider.

**Implementation costs** were not consistently recorded during the trial, as the provider was already active in some hospitals and integration requirements varied. As a result, implementation costs could not be directly derived from the study and were instead based on current provider contracts, which estimate a one-time investment cost of €9900 per hospital. To enable cost comparison across models, implementation costs were converted to per-patient values by dividing €9900 by the mean annual case volume of procedures per hospital (473), yielding an estimated per-patient implementation cost of €20.93.

Combining personnel time, licensing, and implementation costs, the total estimated per-patient costs varied across models (see Table 1).

**Table 1:** Estimated per-patient costs by cost component and implementation model (rounded to full Euro)^a^

Cost component	Trial setting	Hybrid care model	Automated care model
Personnel (minutes × €0.59)	€37 (62 min)	€12 (21 min)	€6 (10 min)
Licence fee (per patient)	€100	€100	€100
Implementation^b^ (per patient)	€21	€21	€21
Total per patient	**€ 158**	**€ 133**	**€ 127**

Bold values indicate the per‑patient totals for each model (sum of personnel, licence, and implementation costs).

^a^Source: Own calculations based on trial time-and-task data, German nursing wage rates, software-provider quotes (licence and implementation), and EPRD Annual Report 2024.

^b^Implementation costs calculated as €9900 per hospital, divided by a mean volume of 473 procedures/year. All values rounded for clarity.

Based on this modelling, the estimated cost per patient is €158 in the Trial Setting, €133 in the Hybrid Care model, and €127 in the Automated Care model.

In Step 4, ‘Population-level outcomes and costs’, to estimate the population-level impact of national-scale implementation, a decision tree model was developed using patient-level data from the *PROMoting Quality* trial. The model reflected the trial design, with branches for the intervention and control arms and outcome paths indicating whether patients met the MCID for health status at 12 months.

**Fig. 3:**
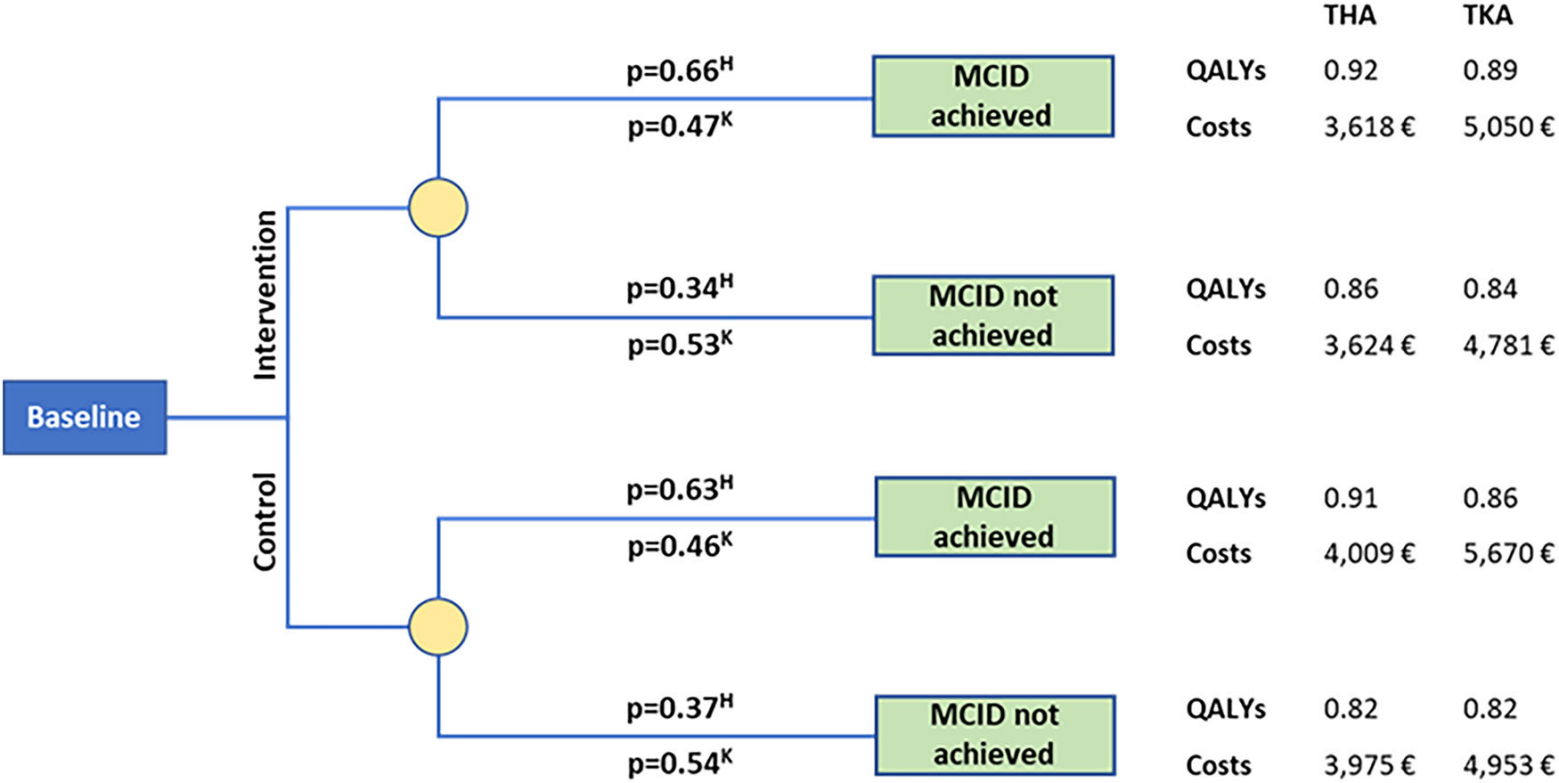
Decision tree model illustrating population-level projections for hip and knee replacements. The diagram shows the transition probability (*p*) of achieving an MCID in health status 12 months after surgery, for patients in the intervention and control groups. Probabilities p^H^ and pK denote the probabilities of patients undergoing hip and knee replacement, respectively. Outcomes are presented separately for total hip arthroplasty (THA) and total knee arthroplasty (TKA), with associated QALYs and costs per patient. Transition probabilities were derived from the PROMoting Quality trial.

Outcomes for each path were expressed in QALYs, derived from EQ-5D-5L utility scores reported at 12 months. Utility weights were based on the German EQ-5D-5L value set, and one-year QALYs were estimated by multiplying the 12-month utility by the time horizon. Scores ranged from 0 (equivalent to death) to 1 (optimal health). Transition probabilities were based on the proportions observed in the trial (see Fig. 3).

Costs were assigned to each outcome using claims data from a subsample of 1038 patients. Specifically, only the 12-month follow-up health expenditures from insurance claims were modelled, while intervention costs were incorporated separately in the next step.

On average, 66% of hip replacement patients and 47% of knee replacement patients in the intervention group achieved an MCID, compared with 63% and 46% in the control group, respectively. Across both procedures, the intervention was associated with better outcomes and lower healthcare costs. For hip replacements, the estimated incremental benefit was 0.023 QALYs with €376 of healthcare expenditure savings per patient; for knee replacements, 0.025 QALYs and the same savings.

To estimate national effects, these per-patient figures were extrapolated from procedure volumes reported in the EPRD Annual Report^21^: 187,640 hip and 155,859 knee replacements in 2023. This corresponds to approximately 4316 additional QALYs for hip-replacement patients and 3896 for knee-replacement patients, alongside projected healthcare expenditure savings of €70.6 million and €58.6 million, respectively.

When intervention costs were incorporated, net savings remained substantial. Depending on the implementation model, total healthcare expenditure savings for hip replacements ranged from €40.9 million to €46.7 million, and for knee replacements from €34.0 million to €38.8 million (see Table 2).

**Table 2:** Population-level outcomes and costs of implementation models^a^

Indication	Group	MCID	p	QALY	Cost^b^	E(Q)	E(C)^b^	∆ QALYs	∆ Healthcare expenditure savings p.p.	QALYs gained p.a.^c^	Healthcare expenditure savings p.a. ^c^
Hip	IG	Yes	0.66	0.92	3618 €	0.90	3620 €	0.023	FU: −376€ TS: −218€ HC: −243€ AC: −249€	4316	FU: −70,552,640€
		No	0.34	0.86	3624 €						TS: −40,905,520€
	CG	Yes	0.63	0.91	4009 €	0.88	3996 €				HC: −45,596,520€
		No	0.37	0.82	3975 €						AC: −46,722,360€
Knee	IG	Yes	0.47	0.89	5050 €	0.86	4907 €	0.025	FU: −376€ TS: −218€ HC: −243€ AC: −249€	3,896	FU: −58,602,984€
		No	0.53	0.84	4781 €						TS: −33,977,262€
	CG	Yes	0.46	0.86	5670 €	0.84	5283 €				HC: −37,873,737€
		No	0.54	0.82	4953 €						AC: −38,808,891€

*MCID* minimal clinically important difference (achieved), *p* transition probabilities, *QALY* quality-adjusted live years, *E(Q)* expected QALYs, *E(C)* expected cost, *ICER* Incremental Cost Effectiveness Ratio, *p.a.* per annum, *p.p.* per patient, *IG* Intervention group, *CG* Control group, *FU* Only follow-up costs without intervention costs, *TS* Trial Setting with intervention costs = €158, *HC* Hybrid Care model with intervention costs = €133, *AC* Automated Care model with intervention costs = €127.

^a^Source: Own calculations using patient-level data from the PROMoting Quality trial (transition probabilities and EQ-5D-5L utilities), statutory health-insurance claims data (12-month expenditures), time-and-task analyses from participating hospitals (personnel inputs), provider cost quotations (licence and implementation), and national case volumes from the EPRD Annual Report 2024.

^b^Considering only 12-month follow-up costs without intervention costs as derived in the model of Fig. 3.

^c^Assuming 187,640 annual primary total hip and 155,859 total knee arthroplasties (following EPRD Annual Report 2024).

Sensitivity analyses evaluated differences in incremental health outcomes and costs across the three implementation models when key cost inputs were varied. In all variations, the intervention remained more effective and less costly than usual care.

#### Licence-fee variation (i)

Reducing the per-patient licence fee from €100 to €20 lowered total per-patient costs to €78 in the Trial Setting, €53 in the Hybrid Care model, and €47 in the Automated Care model (Supplementary Table S3). At the national scale, estimated healthcare expenditure savings increased to €56–62 million for hip replacements and €46–51 million for knee replacements (Supplementary Table S7).

#### Hospital-volume variation (ii)

Implementation costs were recalculated by hospital procedure-volume category. Assumed mean annual volumes of approximately 95, 329, and 1000 procedures for small, medium, and large hospitals yielded per-patient implementation costs of €104, €30, and €10, respectively (Supplementary Table S4). When applied to the implementation models, total per-patient costs ranged from €147 to €241 in the Trial Setting, from €122 to €216 in the Hybrid Care model, and from €116 to €210 in the Automated Care model (Supplementary Table S5).

Because implementation costs vary with procedure volume, national extrapolations were stratified by hospital size. High-volume hospitals (>500 primary procedures/year) accounted for 62% of cases reported in the quality report cards (116,337 hip and 96,633 knee replacements in 2023). In contrast, low-volume hospitals (<200/year) accounted for 6% of hip and knee replacements (11,258 and 9352, respectively). Applying these proportions to the EPRD 2023 totals, and restricting implementation to high-volume hospitals, yielded 2676 additional QALYs for hip patients and 2416 for knee patients, with healthcare expenditure savings of €27–€30 million and €22–€25 million, respectively *(*Supplementary Table S8*)*.

#### Combined variation (iii)

Combining a reduced licence fee (€20 per patient) with high-volume implementation costs (€10 per patient) reduced total per-patient costs to €67 in the Trial Setting, €42 in the Hybrid Care model, and €36 in the Automated Care model (Supplementary Table S6). The estimated healthcare expenditure savings in this best-case analysis ranged from €36 to €40 million for hip replacements and from €30 to €33 million for knee replacements (Supplementary Table S9).

Table 3 synthesises the national impact across the three implementation models (Trial Setting, Hybrid Care, and Automated Care) by reporting annual incremental QALYs and healthcare expenditure savings relative to usual care across four comparative setups. The primary analysis applies a €100 licence fee with mean implementation costs. A licence-fee variation replaces this with a €20 per-patient charge to illustrate price sensitivity. A hospital-volume variation restricts coverage to centres performing more than 500 procedures per year (62% of national procedures) to reflect scale effects. A combined variation applies both high-volume coverage and a €20 licence fee.

**Table 3:** National impact by implementation model and sensitivity analyses^a^

Implementation model	Main analysis		Sensitivity analyses (i)–(iv) with parameter variation							
			(i) Licence-fee variation		(ii) Hospital-volume variation		(iii) Combined variation		(iv) QALY variation	
	∆ QALYs	∆ Health-expenditure savings (€M)	∆ QALYs	∆ Health-expenditure savings (€M)	∆ QALYs	∆ Health-expenditure savings (€M)	∆ QALYs	∆ Health-expenditure savings (€M)	∆ QALYs	∆ Health-expenditure savings (€M)
**Trial setting**	8212	−74.9	8212	−102.4	5092	−48.8	5092	−65.8	8212	−74.9
**Hybrid care model**	8212	−83.5	8212	−111.0	5092	−54.1	5092	−71.1	8212	−83.5
**Automated Care model**	8212	−85.5	8212	−113.0	5092	−55.4	5092	−72.4	4106	−85.5

Main analysis: Columns show Δ QALYs gained per annum and cost savings per annum (€M) versus usual care.

(i) Licence-fee variation: Same as in the main analysis, but licence €20 per patient; complete national volumes. QALYs are the same as in the main analysis for each model; differences reflect costs only.

(ii) Hospital-volume variation: Coverage restricted to 62% of national volumes (>500 procedures/year; hip 116,337; knee 96,633). Licence €100 per patient; implementation cost €10 per patient. Total QALYs reflect 62% coverage (5,092).

(iii) Combined variation: Same coverage as (ii); licence €20 per patient; implementation cost €10 per patient. QALYs are the same as in (ii); differences are cost only.

(iv) QALY variation: QALY gains are reduced in the Automated Care model in proportion to reduced human involvement. All other parameters are as in the main analysis.

General notes: QALYs from EQ-5D-5L utilities at 12 months (German value set); hip and knee aggregated. Cost savings reported from the statutory health insurance perspective over 12 months; no discounting. Monetary values rounded to €0.1 M.

^a^Source: Own calculations based on patient-level data from the PROMoting Quality trial (transition probabilities and EQ-5D-5L utilities), statutory health-insurance claims data (12-month costs), time-and-task analyses (personnel inputs), provider quotations (licence and implementation costs), and national procedure volumes from the EPRD Annual Report 2024.

QALYs are aggregated across hip and knee procedures. In the all-hospitals baseline and the licence-fee variation, only cost inputs vary while clinical transition probabilities and utility values are held constant. Per-patient QALY gains and total QALYs are therefore identical across implementation models. In the hospital-volume and combined variations, coverage is restricted to a subset of hospitals. Per-patient QALY gains remain unchanged, but total QALYs scale with the share of national procedures included (8212 at 100% coverage versus 5092 at 62%). Differences in total QALYs reflect coverage only, as the per-patient health benefit (0.023 to 0.025 QALYs) is constant across all variations.

#### QALY variation (iv)

The potential variation in effectiveness was assumed to arise from differences in follow-up procedures across implementation models. In the Hybrid Care model, follow-up interactions were supported by study assistants, whereas these interpersonal components were largely absent in the Automated Care model. As shown in Fig. 2, supported follow-ups accounted for approximately half of the observed time differential between models (21 versus 10 min per patient). Interpersonal contact during follow-ups was assumed to contribute to patient engagement and the perceived usefulness of feedback, with potential implications for improvements in HRQoL. To examine this assumption, a health-benefit variation was modelled in which the incremental QALY gains were reduced by half relative to the base-case estimates (0.012 for hip and 0.013 for knee replacements), in proportion to the reduction in human involvement. At the same time, all other costs and transition parameters were retained at base-case levels. Despite reduced health benefits, the intervention remained cost-saving across all implementation models, indicating that, even under conservative assumptions of diminished effectiveness, the PROM-based intervention continues to yield economic and clinical benefits at a national scale (Supplementary Table S10).

These findings highlight two cost levers: licence pricing and hospital volume. Reducing the licence from €100 to €20 increases annual savings in national healthcare expenditure by around €27.5 million at full coverage across implementation models, with QALYs unchanged at 8212. Restricting PROM collection to high-volume hospitals (62% of cases) reduces the achievable QALYs from 8212 to 5092, yet still yields annual healthcare expenditure savings of €48.8–€55.4 million. The combined variation preserves 5092 QALYs and increases annual savings by approximately €17 million relative to high-volume alone. Across scenarios, the Automated Care model consistently achieved the lowest costs in high-volume settings. Extending these sensitivity results, the Automated Care model was assessed for changes in the cost–QALY trade-off under reduced human involvement. Assuming health gains are proportional to human participation, QALYs would decrease from 8212 to 4106; despite this reduction, the model retains the same cost-saving potential as the primary analysis, with expected but lower health benefits.

## Discussion

RCTs remain the gold standard for evaluating the clinical and economic impact of new health interventions or technologies^22^. However, their tightly controlled settings often diverge from real-world care delivery, particularly in complex or resource-constrained environments^23^. This gap between research and routine implementation has become especially visible in the field of DHIs, where uptake remains limited despite favourable trial evidence^24,25^.

In this context, earlier studies of the PROMoting Quality trial demonstrated improved patient-relevant outcomes and cost-effectiveness. The present analysis indicates that scaling from the trial to the national level could further increase value (in terms of outcome achieved per cost unit), because economies of scale in PROM collection reduce per-patient costs while maintaining (at least) the same outcome gains. However, real-world adoption has so far been confined to a small number of high-volume, specialised centres. This pattern reflects a broader trend observed in Germany’s PROM-based quality initiatives, such as the PROvalue Endo contract, which has primarily been implemented in hospitals already equipped with the necessary infrastructure, case volume, and digital capabilities to support continuous outcome monitoring. As national PROM mandates, such as the EPRD expansion, move toward broader rollout, this selective uptake raises questions about scalability, equity and the resources needed for integration into routine care.

A structured four-step extrapolation was undertaken using data from the PROMoting Quality trial. This approach identified the core components of the intervention, modelled varying levels of personnel involvement and automation in two implementation models, and projected costs and outcomes at the national scale. The primary contribution lies in the implementation perspective: translating trial evidence into operational requirements, identifying resource drivers, and modelling scalability under varying assumptions. The favourable cost-effectiveness observed under base-case assumptions should therefore be interpreted as indicative of the intervention’s potential to improve outcomes while reducing costs, rather than as a definitive economic conclusion. Across implementation models, the intervention was projected to save costs and improve health outcomes, including those with reduced personnel time requirements: from 62 min per patient in the trial to 21 min in the Hybrid Care model and 10 min in the Automated Care model. When extrapolated nationally, this translated into more than 8000 additional QALYs and annual savings of €86 million in healthcare expenditure. Using the 2023 EPRD primary case volumes (187,640 total hip and 155,859 total knee arthroplasties) and the adjusted post-operative cost estimates from Schöner et al.^17^, this corresponds to approximately €129 million in total annual savings on post-operative expenditure, equivalent to 8.2% of the EPRD-participating hospitals’ post-operative costs, which sum to approximately €1.57 billion (THA: €749.9 million; TKA: €823.4 million).

These findings should be interpreted as projections from an exploratory modelling exercise rather than as definitive estimates of real-world cost-effectiveness. While these projections suggest substantial potential, they rely on modelling assumptions that may not fully reflect the realities of routine care. Critically, the analysis assumed that trial effects would transfer to the streamlined implementation models, although reduced human interaction may attenuate these effects. For example, it remains unclear whether and to what extent the observed effects are attributable to study conditions, including the close support provided by study assistants. In the trial’s control group, intended to represent standard care, patients also completed PROMs at admission and discharge, which may have led to benefits such as self-reflection or a sense of being monitored. Hence, this actual ‘standard of care plus’ group may have led to a ‘trial placebo effect,’ narrowing the observed difference between groups. A recent systematic review and meta-analysis by Long et al.^26^ on the *subtle intervention* effect of PROMs supports this interpretation, showing that repeated PROM completion can influence patient perceptions and behaviours even in the absence of any active follow-up. This implies that the true potential of the intervention, relative to real-world control conditions, may be even greater than observed in the trial.

At the same time, the intervention’s observed outcomes likely depended not only on data collection but also on its interpersonal components. Steinbeck et al.^16^ described a ‘caring effect’ in which patients may have felt reassured and engaged through structured feedback based on their PROMs and personnel-initiated contact by study assistants. This was particularly meaningful as the trial was conducted during the COVID-19 pandemic, when remote interactions helped compensate for reduced in-person care^27^.

This behavioural mechanism also highlights a key implementation trade-off: in the trial, 70% of PROM alerts resulted in follow-up contact, although only a minority required physician escalation. These low-intensity follow-up interactions appear central to sustaining patient engagement, even when clinical escalation was not needed. The Automated Care model was designed to maximise efficiency by removing most interpersonal elements. However, doing so may risk undermining the behavioural mechanisms that contributed to the observed effects.

Related literature supports caution. Gomes, Murray & Raftery^15^ emphasise that DHIs evolve through frequent content updates and scaling, which can change resource use and benefits; accordingly, economic and effectiveness evaluations should be revisited when such changes occur rather than assuming trial effects will persist unchanged. Similarly, Kowatsch et al.^28^ recommend reiterating the design and evaluation phases whenever updates alter the intervention’s causal chain, underscoring that post-implementation effects may diverge from trial findings and therefore warrant renewed assessment.

The Hybrid Care model represents a pragmatic compromise. It reduces personnel time to one-third of the trial level while preserving brief but meaningful human contact during onboarding and follow-up. Prior research confirms that consistency of use in self-monitoring interventions is a key determinant of intervention success^29^. However, digital tools that lack interpersonal interaction are prone to declining engagement^30,31^ and often exhibit high dropout rates, often due to usability challenges or insufficient integration into patients’ daily lives^32^.

To mitigate these risks, minimal but meaningful feedback mechanisms should be preserved, for instance, through digital coaches or automated follow-up alerts, which can help sustain patient engagement and maintain trust in the intervention^19^. Hybrid PROM collection models have demonstrated higher response rates than fully automated ones^33^. In light of this, the Hybrid Care model may preserve the behavioural mechanisms observed in the trial—such as the ‘caring effect’—while reducing personnel time and maintaining resource efficiency.

Importantly, the presented extrapolation analysis assumed that intervention effects on outcomes and costs were consistent across all scenarios. The estimates were based on the assumption that trial-level effectiveness (QALY gains of 0.023 to 0.025 and cost savings of €218 to €376 per patient) would remain consistent across scenarios despite varying levels of interpersonal contact. While maintaining such effectiveness is plausible in the Hybrid Care model, it is less certain in the Automated Care model, where the absence of interpersonal contact may reduce engagement and, in turn, (cost-)effectiveness.

Although the available data did not allow for isolating causal pathways, it is unlikely that the intervention effect stems solely from interpersonal contact. A positive, albeit potentially smaller, effect is still expected even with reduced personnel input. Moreover, even if QALY gains were substantially smaller or approached zero, the intervention would remain cost-saving in both implementation models. This was demonstrated in the sensitivity analysis (iv), which reduced the assumed QALY gains in proportion to the reduced human involvement. Even with marginal health gains, substantial cost savings remain. Nevertheless, the hybrid model is the most balanced, offering greater QALY gains and substantial cost-saving potential.

From a health system perspective, successful implementation requires more than demonstrating clinical effectiveness and economic advantages. Real-world feasibility depends on integration into existing infrastructures, workflows, and workforce capacity^34^. As hospitals face workforce shortages^35^, the implementation of scalable solutions hinges on resource-efficient delivery. Additional digital tasks without adequate support risk overburdening clinical teams^13^.

Implementation costs, such as software integration and training, may decrease with scale but remain substantial, particularly in low-volume hospitals. Grimberg et al.^21^ note that hospitals performing fewer joint replacement procedures incur disproportionately higher per-patient costs, underscoring the financial challenge of introducing such interventions in low-volume settings. Additionally, Rombey et al.^36^ demonstrate that such variation in uptake is not solely a function of economic factors, but is also strongly associated with institutional research involvement, available resources, and digital maturity. This reinforces the point that implementation success hinges not only on the intervention’s design, but also on the readiness and capacity of adopting institutions.

The primary analysis, under conservative assumptions, showed that implementation costs and licence fees are the principal cost drivers. Sensitivity analyses indicate that modifying these levers—restricting adoption to high-volume centres or lowering licence fees—materially increases potential savings. By contrast, higher per-patient implementation costs make uptake less attractive in hospitals with low patient volumes. Quality considerations also favour centralisation: re-revision rates after total knee arthroplasty are higher in low-volume hospitals, with consistently better outcomes in high-volume centres^37^.

Finally, the PROMoting Quality trial raises broader policy questions. Despite public funding, there is currently no national strategy in Germany to ensure that effective interventions are implemented after positive evaluation. As Lindemann et al.^38^ argue, implementation should be recognised as an integral phase of the research cycle, especially when results indicate scalable, patient-centred, and cost-effective innovations. Without dedicated structures and incentives for adoption, even well-substantiated innovations may fail to generate their intended impact in routine care settings.

This analysis has limitations. Estimates rely on a 12-month horizon, using EQ-5D-5L utilities at 12 months, then scaled by 1 year. This assumes a constant recovery trajectory and may overlook variation in recovery speed. Trial effects were applied across implementation models without model-specific re-estimation; accordingly, per-patient effect sizes may attenuate under more automated delivery with reduced human interaction. Model input uncertainty is an additional limitation, as time estimates were derived from structured interviews rather than direct observation and may therefore be affected by recall or reporting bias. Although deterministic sensitivity analyses examined key cost and effectiveness parameters, full probabilistic uncertainty analysis was beyond the scope of this exploratory modelling approach. The control arm’s PROM completion may have induced measurement effects, thereby reducing observed between-group differences. Claims analyses used a consented subsample and multiple imputation under a missing-at-random assumption; selection and imputation bias cannot be excluded. National extrapolations utilised hospital-size strata and their corresponding proportions from the national quality reports, which were pragmatically applied to EPRD case totals. Discrepancies between report coverage and the voluntary EPRD may bias volume weighting. Economic results reflect a statutory insurance perspective over 12 months, with no discounting; provider-side expenditures for the intervention are excluded from payer costs. Workflow feasibility (onboarding, alert routing, interoperability) was not experimentally varied and requires site-level validation.

Several practical and conceptual challenges must be addressed to sustainably scale PROM-based monitoring. In the trial, forwarding PROM alerts to treating physicians was often impractical due to outdated communication methods, such as fax or postal mail. Future implementation must establish standardised, secure interfaces that seamlessly link hospitals, outpatient providers, and patients. Moreover, precise time tracking (e.g. time stamps) of individual intervention steps could provide robust estimates of the associated personnel requirements, enabling more accurate estimations for workforce planning.

Both site personnel and patients require structured training to ensure familiarity with digital PROMs. To complement training, a project in Germany (‘MIA-PROM’) is piloting a multimodal, AI-supported assistant that guides patients through PROM completion and follow-up using natural conversation, speech, and visual prompts. Future projects of this kind may provide evidence on feasibility and acceptability in routine care, including whether a caring effect can be reproduced with minimal personnel input.

In conclusion, this study translates trial-based evidence on PROM-based monitoring after joint replacement into a structured assessment of implementation requirements and potential system-level impact in Germany. The findings suggest that streamlined delivery models may substantially reduce resource use while maintaining favourable cost and outcome profiles under base-case assumptions. At the same time, these projections remain contingent on key assumptions, particularly regarding the transferability of trial effects to routine care and the level of patient engagement under more automated models.

While the study offers plausible hypotheses about mechanisms through which the intervention might work, these remain speculative. Future research should assess real-world effectiveness, patient engagement, and system integration under routine care conditions, while also testing individual components in isolation, such as PROM completion, alert triggering, or follow-up contact. Such component analysis could help validate and optimise mechanisms of action, eliminate redundant steps and improve efficiency.

## Methods

*PROMoting Quality* was a multicentre RCT conducted across nine German hospitals between 2019 and 2022. It evaluated a digital PROM-based monitoring intervention for patients undergoing elective primary hip or knee arthroplasty. In both trial arms, patients received standard care and completed PROMs at admission, discharge, and 12 months post-surgery. In the intervention group, patients additionally completed PROMs at 1, 3, and 6 months, with automated alerts generated when responses indicated atypical recovery. Study personnel reviewed alerts, initiated follow-up contact, and, if needed, referred patients back to their treating physicians. The published protocol provides further detail^14^. The trial was conducted in accordance with the Declaration of Helsinki. It was approved by the Charité’s Ethics Committee, Berlin (EA4/169/19), and registered with the German Register of Clinical Trials (DRKS) under DRKS00019916 on 26 November 2019. Each patient gave informed consent to participate in the study.

The analysis drew on data from 6807 patients, including demographic information, treatment details, and PROM results. HRQoL was measured using the EuroQol Five Levels Five Dimensions (EQ-5D-5L) and EuroQol Visual Analogue Scale (EQ-VAS); joint-specific physical function with the Hip Disability and Osteoarthritis Outcome Score—Physical Function Short Form (HOOS-PS) and Knee Injury Osteoarthritis Outcome Score—Physical Function Short Form (KOOS-PS); and mental health with PROMIS Depression and Fatigue short forms. Insurance claims data were available for 1038 patients insured with 24 statutory health insurance funds, covering inpatient and outpatient care, prescriptions, remedies, and medical aids during the 12 months after surgery.

### Extrapolation framework

The intervention was deconstructed into operational components to assess feasibility for routine care. Activities specific to the research setting (e.g. study enrolment procedures, trial documentation) were identified and excluded. The remaining components were reviewed for their resource requirements and implementation costs. The extrapolation estimated costs and effects for broader patient populations by retaining only elements essential to the core intervention and eliminating study-specific procedures.

The extrapolation of the main analysis followed four structured steps based on the framework by Murray et al.^39^:

For Step 1, ‘Time and task analysis’, tasks undertaken by study assistants were mapped, and the average time required per task (in minutes) was obtained through structured interviews conducted across participating hospitals (see Supplementary Material Table S1). Tasks were classified as either study-specific or required for routine PROM-based monitoring.

In Step 2, ‘Streamlined implementation models’, study-only activities were removed, and two delivery models were specified, differing in the degree of automation and human involvement based on the Step-1 time estimates.

For Step 3, ‘Scalability’, national-level intervention costs were projected for three categories:

- **Personnel costs**, derived from estimated time inputs and standard nursing wage rates in Germany (see Supplementary Material Table S2).
- **Licence fees** charged per patient for PROM user licences, and continued software support and updates, with projections provided by the trial’s software provider.
- **Software implementation costs** consisting of one-time investment costs borne by the hospitals, including installation, maintenance, personnel training, and modifications specific to the study design. Routine care costs were projected in collaboration with the software provider. To determine implementation costs at the patient level, the average number of hip and knee replacement procedures per hospital was calculated using the latest registry data. According to the 2024 EPRD Annual Report^21^, a total of 343,499 primary (187,640 hip and 155,859 knee) arthroplasty procedures were reported in 2023 by 726 participating hospitals, resulting in an average annual case volume of approximately 473 procedures per hospital.

In Step 4, ‘Population-level outcomes and costs’, a decision tree model was developed to extrapolate the intervention’s national impact. The model distinguished between intervention and control arms for hip and knee replacement procedures and assessed whether patients met the MCID in EQ-5D-5L scores between admission and 12 months^40^. Transition probabilities were derived from trial data, with four outcome paths linked to distinct cost and health utility values.

To facilitate cross-country comparisons and enable population-level projections, expected outcomes at each decision-tree endpoint were expressed as QALYs. QALYs were calculated using EQ-5D-5L utility scores obtained from the 12-month follow-up survey. Utility weights were derived from the German EQ-5D-5L value set. One-year QALY estimates were computed by multiplying the 12-month utility value by the one-year period, applying the area-under-the-curve method based on a single time point. Scores ranged from 0 (death) to 1 (optimal health).

Healthcare expenditure reflected 12-month claims. Missing data were addressed using multiple imputation by random forests, under the assumption of missing-at-random. No discounting was applied, given the one-year horizon. Incremental health outcomes and costs were calculated as the differences between the intervention and control groups.

### Sensitivity analyses

Given the exploratory nature of the extrapolation, all estimates used are associated with uncertainty that requires adequate quantification. Uncertainty was assessed using structured deterministic sensitivity analyses, which examined the impact of varying cost and outcome components on the results, applied to all implementation models:

(i) **Licence-fee variation:** A reduced per-patient licence fee of 20€ per patient based on automated PROM collection in Pronk et al.^33^.
(ii) **Hospital-volume variation:** Implementation costs were recalculated by hospital procedure volume. Hospitals were categorised into three groups (0–200, 201–500, and >500 annual primary hip and knee arthroplasty procedures) using data from the German national hospital quality report cards. These thresholds approximate the categories used in the EPRD Annual Report; because EPRD participation is voluntary and reported case numbers are lower than those in the national quality reports, the thresholds were applied as a pragmatic approximation for modelling purposes. Per-patient implementation costs were calculated by dividing hospital-level implementation costs by the mean annual procedure volume within each category, and then aggregated into a distribution-adjusted national estimate by weighting each category by its proportion from the quality reports. Personnel-time inputs and software licence-fee assumptions were held constant across implementation models.
(iii) **Combined variation**: Best-case combination of reduced licence fees and high-volume hospitals.
(iv) **QALY variation**: Based on the variation in human involvement described in Step 2, an alternative scenario was modelled in which the incremental QALY estimate from Step 4 was adjusted to reflect a lower health gain for the delivery model with the least human input.

For (i)-(iii), only intervention cost parameters were varied; clinical transition probabilities, QALY estimates, and 12-month follow-up costs remained constant at base-case levels. For (iv), only the effectiveness parameter was varied; all cost and transition-probability parameters were held constant. The personnel time component is also associated with uncertainty, as the underlying estimates were derived from a survey of project-involved study assistants and supplemented by input from the software provider. However, because variation in the reported time inputs was minimal and no comparable empirical data were available, there was no robust basis for specifying plausible parameter ranges for this cost component. In addition, variation in personnel requirements is already partly captured in the main analysis through the different care scenarios. Accordingly, no separate sensitivity analysis was conducted for personnel time. The reported estimates should therefore be interpreted as exploratory approximations that may vary across routine-care settings depending on local process structures.

## Supplementary information

Electronic Supplementary Material.

## Data Availability

The patient‑level datasets generated and/or analysed during this study contain personal health information and are protected under German data‑protection regulations. As a result, they cannot be shared publicly. De‑identified data necessary to replicate the analyses are stored on secure institutional servers at Technische Universität Berlin and can be made available to qualified researchers on reasonable request to the corresponding author, subject to a data‑use agreement.

## Code availability

All quantitative analyses were performed in Stata 17 MP using standard statistical commands. No custom algorithms or external software packages were developed. The analysis scripts used for data preparation, cost-estimation, and extrapolation modelling are stored on secure institutional servers at Technische Universität Berlin. Because the scripts reference patient-level identifiers, they cannot be made publicly available. Access to these scripts may be provided to qualified researchers for verification purposes upon reasonable request to the corresponding author and approval of a data-use agreement.
