# Supplementary material for: National scale up of PROM based monitoring after joint replacement in Germany
Source: Npj Health Syst. 2026 Jul 8;3:63. doi: 10.1038/s44401-026-00122-y (PMC13354145; doi:10.1038/s44401-026-00122-y)
Supplement: Supplementary file 1 — Electronic Supplementary Material. [file 44401_2026_122_MOESM1_ESM.pdf]

Electronic Supplementary Material

Article title: National Scale Up of PROM Based Monitoring After Joint Replacement in Germany

Table of Contents

1. Structured Interviews 2
2. Personnel Cost Calculation........ 4
3. Sensitivity Analysis (i) – Reduced licence fee 5
4. Implementation Costs per Patient by Hospital Volume 5
5. Sensitivity Analysis (ii) – Per Patient Costs by Model and Hospital Volume 6
6. Sensitivity Analysis (iii) – Combined variation 6
7. Sensitivity Analysis (i) – Impact of Reduced Licence Fee on Costs, QALYs and ICERs 7
8. Sensitivity Analysis (ii) – Total Costs by Model and Hospital Volume........ 8
9. Sensitivity Analysis (iii) – Reduced License Fee and High Hospital Volume........ 9
10. Sensitivity Analysis (iv) of Costs, QALYs, and ICERs........ 10

1. Structured Interviews

Table S1. Structured interviews conducted across participating hospitals (infrastructure, procedures, and time requirements)

**Questions about infrastructure and procedure**
Which clinic do you belong to?
What training do you have, or are you a trained study assistant?
Did you already have professional experience with PROM studies before PROMoting Quality?
How many study assistants work full-time for the project during the patient follow-up phase?
How many study assistants work part-time for the project during the patient follow-up phase?
Do you have a room where you can conduct patient recruitment or follow-up (alone) (e.g. reception room)?
Where in the facility is your reception room located?
Did you have contact with the (study) physicians during the recruitment phase? If yes, how often?
Do you contact patients by telephone to assist them in answering the questionnaires in the follow-up?
Was the project flyer displayed in your clinic and if so, where?
Do you feel that the flyer was useful?
Were the iPads permanently installed in your clinic and if so, where?
Were there any changes to the iPad location during recruitment?
In your opinion, what were the biggest organizational hurdles in patient recruitment? (apart from the restrictions due to the pandemic; multiple answers possible)
When and how did inclusion in the study usually take place?
Were there any changes in patient inclusion after the pandemic?
Did you find out in advance whether the patient met the inclusion criteria for the study and if so, how?
How did you find out when the patient left the clinic?
Were there or are there hurdles in coordinating with colleagues (e.g. ward physicians) in the project?
Are there any obstacles to contacting patients during their stay in the clinic?
Are there any obstacles to contacting patients after their hospital stay?
Were there or are there barriers to contacting the doctor providing further treatment?
Were there or are there other hurdles in the project and how did you address them?
What were the biggest hurdles you encountered during inclusion from the patient's perspective? (please select a maximum of 3 answers)
Approximately how many patients were you unable to recruit to the study even though they met the inclusion criteria?
How many patients who would otherwise have met all inclusion criteria could not be included in the study due to a lack of e-mail access?

How many patient discharges did you miss because you were not informed about the discharge?
Were all days of the week (Mon-Fri) covered by the presence of at least one study assistant?
If no: How many days per working week (Mon-Fri) was no study assistant present in the clinic?
Which aspects of the recruitment phase do you remember particularly positively – and which not? Please elaborate.
Do you have any further remarks/comments on the infrastructure and procedure?

**Questions about the implementation of the study design**
Did you have continuous access to the patient data or to the HIS? Can you specify in which months this was not the case?
How was the patient informed in your clinic? Did you have any difficulties with this?
How many weeks/months before the procedure did you inform the patient?
Did you give the patient a copy of the consent form?
Did you/your clinic send the flyer to patients in advance?
Did you feel that you were able to respond well to queries from the patient (e.g. about data protection, questionnaires, if thresholds were exceeded)? If not, what was the reason for this?
Did you conduct the initial interview together with the patient?
Did you conduct the discharge survey together with the patient?
Did you conduct the follow-up interview together with the patient?
Did the patient receive their evaluation form (e.g. on discharge?)
How often did you conduct the follow-up survey together with the patient?
Did you make use of the PROMoting Quality Guidelines when contacting patients when thresholds were exceeded?
How did you usually proceed when a threshold was exceeded? (e.g. did you first contact the patient by e-mail before calling them; did you explicitly address critical values; did you suggest to the patient, for example, that they visit the doctor providing follow-up treatment, etc….)
In your opinion, were the telephone calls to patients after the threshold was exceeded helpful for the patient?
If you made phone calls to the patient after the threshold was exceeded, how often did the patient report problems in the operated joint?
How did the follow-up physician receive the study information?
Did any medical colleagues other than yourself have contact with the patient when the threshold was exceeded?
The post-treatment physician was informed about increased threshold values…
How are/were the clinicians involved?
How do you feel about the doctors' acceptance of being involved in the study?

Do you have any remarks/comments on the implementation of the study design?

**Questions about the time required**
Average time required for admission (informing the patient, obtaining signature)
Average time required for the initial consultation (e.g. support with queries)
Average time required for follow-up questioning (e.g. telephone support for queries)
Average time spent when threshold values are exceeded (e.g. through telephone support for queries and notification of the doctor providing follow-up treatment)
Do you have any remarks/comments on the time spent?

2. Personnel Cost Calculation

Table S2. Calculation and pricing of personnel minutes in Germany

Working days per year	365
- weekends	104
- training days	5
- holidays	30
- national holidays (Berlin)	7
Gross working days per year	219
Hours per working day	8
- Time not on patients	1
Hours per year	1,533 (=219 x 7)
- Absenteeism[1]	20 days = 120 h
Net working hours per year	1,413
**Net working minutes per year**	**84,780**
Mean gross income[2] [€]	39,497
+ EC health insurance[3]	2,883 (7.3 %)
+ EC nursing care insurance[3]	592 (1.5 %)
+ EC unemployment insurance[3]	474 (1.2 %)
+ EC pension insurance[3]	3,673 (9.3%)
+ 13th monthly salary	3,291
**Mean yearly income [€]**	**50,410**
**Costs [€] per working minute**	**0,59**

Legend: EC = Employer contribution

1 Source: AOK Fehlzeitenreport

2 Source: Bundesagentur für Arbeit

3 Source: Sozialversicherungsbeiträge 2021

3. Sensitivity Analysis (i) – Reduced licence fee

Table S3. Sensitivity analysis (i): Reduced licence fee (€20) - per-patient intervention costs by implementation model

Cost component	Trial Setting	Hybrid Care model	Automated Care model
Personnel (minutes × €0.59)	€37 (62 min)	€12 (21 min)	€6 (10 min)
Licence fee (per patient)	€20	€20	€20
Implementation[1] (per patient)	€21	€21	€21
**Total per patient**	**€ 78**	**€ 53**	**€ 47**

Legend: Licence fee variation calculated with a reduced licence fee of €20 per patient (based on Pronk et al., 2019). Implementation cost per patient uses the base-case approach from the Endoprothesenregister Deutschland (EPRD) Jahresbericht 2024 mean volume (473 procedures per site and year). Personnel time was held constant at 62 minutes (Trial Setting), 21 minutes (Hybrid Care model), and 10 minutes (Automated Care model).

[1] Implementation costs per patient calculated as €9,900 per hospital divided by mean annual cases.

4. Implementation Costs per Patient by Hospital Volume

Table S4. Implementation cost per patient by hospital procedure volume

Category	Number of hospitals	Mean annual cases	Share of hospitals	Share of total cases	Implementation cost per patient
0-200	320	~95	29%	6%	€104
201-500	478	~329	43%	32%	€30
>500	304	~1,000	28%	62%	€10

Legend: Hospital procedure volume variation calculated using hospital procedure volumes from the 2023 German hospital quality report cards (n = 1,102; total cases = 491,951). Categories (0-200, 201-500, >500 annual procedures) approximate the grouping in the German Arthroplasty Registry (EPRD) Annual Report (2024). Per-patient implementation costs were estimated by dividing the one-time implementation cost per hospital by the mean annual volume within each category. Shares of hospitals and cases refer to all sites reporting primary hip or knee arthroplasty in 2023. Implementation costs per patient calculated as €9,900 per hospital divided by mean annual cases.

5. Sensitivity Analysis (ii) – Per Patient Costs by Model and Hospital Volume

Table S5. Sensitivity analysis (ii): Per-patient intervention costs by implementation model and hospital procedure volume

Cost component	Trial Setting	Hybrid Care model	Automated Care model
Personnel (minutes × €0.59)	€37 (62 min)	€12 (21 min)	€6 (10 min)
Licence fee (per patient)	€100	€100	€100
Implementation (per patient)	€10 - €104	€10 - €104	€10 - €104
**Total per patient**	**€ 147 - € 241**	**€ 122 - € 216**	**€ 116 - € 210**

Legend: Calculated with personnel time as observed in the trial (62/21/10 minutes for Trial Setting/Hybrid Care model/Automated Care model), a €100 licence fee per patient, and implementation costs per patient ranging from €10-€104 derived from the three hospital-volume categories in Table S4. Totals are shown as ranges corresponding to the lowest- vs. highest-volume categories; these ranges do not incorporate national distribution weights.

6. Sensitivity Analysis (iii) – Combined variation

Table S6. Sensitivity Analysis (iii): Combined variation (reduced licence fees and high-volume hospitals)

Cost component	Trial Setting	Hybrid Care model	Automated Care model
Personnel (minutes × €0.59)	€37 (62 min)	€12 (21 min)	€6 (10 min)
Licence fee (per patient)	€20	€20	€20
Implementation[1] (per patient)	€10	€10	€10
**Total per patient**	**€ 67**	**€ 42**	**€ 36**

Legend: Calculated as a best-case combination applying a €20 licence fee per patient and the implementation cost per patient from the highest-volume category (>500 procedures per year; €10). Personnel time was held constant at 62/21/10 minutes for Trial Setting/Hybrid Care model/Automated Care model.

7. Sensitivity Analysis (i) – Impact of Reduced Licence Fee on Costs, QALYs and ICERs

Table S7. Sensitivity analysis (i): Reduced licence fee (20€) - impact on costs, QALYs, and ICERs

Indication	Group	MCID	p	QALY	Cost[1]	E(Q)	E (C)[1]	Δ QALYs	Δ Healthcare Expenditure Savings p.p.	QALYs gained p.a.[1]	Healthcare Expenditure Savings p.a.[2]	ICER
Hip	IG	Yes	0.66	0.92	3,618 €	0.90	3,620 €	0.023	FU: -376€	4,316	FU: -70,552,640€	FU: -16,347€
		No	0.34	0.86	3,624 €				TS: -298€		SS: -55,916,720€	SS: -12,956€
	CG	Yes	0.63	0.91	4,009 €	0.88	3,996 €		HC: -323€		HC: -60,607,720€	HC: -14,043€
		No	0.37	0.82	3,975 €				AC: -329€		AC: -61,733,560€	AC: -14,303€
Knee	IG	Yes	0.47	0.89	5,050 €	0.86	4,907 €	0.025	FU: -376€	3,896	FU: -43,458,456€	FU: -11,155€
		No	0.53	0.84	4,781 €				TS: -298€		SS: -46,445,982€	SS: -11,921€
	CG	Yes	0.46	0.86	5,670 €	0.84	5,283 €		HC: -323€		HC: -50,342,457€	HC: -12,922€
		No	0.54	0.82	4,953 €				AC: -329€		AC: -51,277,611€	AC: -13,162€

Legend: MCID = minimal clinically important difference; p = transition probabilities; QALY = quality-adjusted life years; E(Q) = expected QALYs; E(C) = expected healthcare expenditure; ICER = incremental cost-effectiveness ratio; p.a. = per annum; p.p. = per patient; IG = intervention group; CG = control group; FU = follow-up expenditure only (no intervention cost); TS = Trial Setting; HC = Hybrid Care; AC = Automated Care.

Costs in the table's cost columns are 12-month follow-up healthcare expenditure only; TS/HC/AC add the scenario-specific intervention cost per patient. With a €20 licence, intervention cost per patient is: TS €78, HC €53, AC €47 (personnel 62/21/10 min × €0.59 + implementation €21 + licence €20).

ICERs were calculated as the net per-patient cost difference [E(C)IG-E(C)CG] plus the scenario's intervention cost per patient [TS/HC/AC] divided by Δ QALYs. Negative ICERs indicate dominance (more QALYs at lower cost).

[1] *Considering only 12-month follow-up costs without intervention costs*

[2] *Assuming 187,640 annual primary total hip and 155,859 total knee arthroplasties (following Endoprothesenregister Deutschland (EPRD) - Jahresbericht 2024)*

8. Sensitivity Analysis (ii) – Total Costs by Model and Hospital Volume

Table S8. Sensitivity Analysis (ii): Hospital-volume variation - high-volume hospitals (>500 cases/year)

Indication	Group	MCID	p	QALY	Cost[1]	E(Q)	E (C)[1]	Δ QALYs	Δ Healthcare Expenditure Savings p.p.	QALYs gained p.a.[1]	Healthcare Expenditure Savings p.a.[2]	ICER
Hip	IG	Yes	0.66	0.92	3,618 €	0.90	3,620 €	0.023	FU: -376€	2,676	FU: -43,742,637€	FU: -16,348€
		No	0.34	0.86	3,624 €				TS: -229€		TS: -26,641,127€	TS: -9,957€
	CG	Yes	0.63	0.91	4,009 €	0.88	3,996 €		HC: -254€		HC: -29,549,547€	HC: -11,043€
		No	0.37	0.82	3,975 €				AC: -260€		AC: -30,247,568€	AC: -11,304€
Knee	IG	Yes	0.47	0.89	5,050 €	0.86	4,907 €	0.025	FU: -376€	2,416	FU: -36,333,850€	FU: -15,040€
		No	0.53	0.84	4,781 €				TS: -229€		TS: -22,128,861€	TS: -9,160€
	CG	Yes	0.46	0.86	5,670 €	0.84	5,283 €		HC: -254€		HC: -24,544,675€	HC: -10,160€
		No	0.54	0.82	4,953 €				AC: -260€		AC: -25,124,471€	AC: -10,400€

Legend: MCID = minimal clinically important difference; p = transition probability; QALY = quality-adjusted life years; E(Q) = expected QALYs; E(C) = expected healthcare expenditure; ICER = incremental cost-effectiveness ratio; p.a. = per annum; p.p. = per patient; IG = intervention group; CG = control group; FU = follow-up expenditure only (no intervention cost); TS = Trial Setting; HC = Hybrid Care; AC = Automated Care.

Cost columns report 12-month follow-up healthcare expenditure only; TS/HC/AC add the scenario-specific intervention cost per patient.

In this high-volume scenario (licence €100; implementation €10), intervention cost per patient is: TS €147 = personnel €37 (62×€0.59) + implementation €10 + licence €100; HC €122 = €12 + €10 + €100; AC €116 = €6 + €10 + €100.

ICERs were calculated as $[E(C)_{IG} - E(C)_{CG}] +$ *intervention cost per patient (TS/HC/AC)divided by ΔQALYs. Negative ICERs indicate dominance.*

[1] *Considering only 12-month follow-up costs without intervention costs*

[2] *Assuming 62% of 187,640 = 116,337 annual primary total hip and 62% of 155,859 = 96,633 total knee arthroplasties that are performed in the large hospital size category with >500 cases per year (following Endoprothesenregister Deutschland (EPRD) - Jahresbericht 2024)*

9. Sensitivity Analysis (iii) – Reduced License Fee and High Hospital Volume

Table S9. Sensitivity Analysis (iii): Combined variation (reduced licence fee €20 and high-volume hospitals)

Indication	Group	MCID	p	QALY	Cost[1]	E(Q)	E (C)[1]	Δ QALYs	Δ Healthcare Expenditure Savings p.p.	QALYs gained p.a.[1]	Healthcare Expenditure Savings p.a.[2]	ICER
Hip	IG	Yes	0.66	0.92	3,618 €	0.90	3,620 €	0.023	FU: -376€	2,676	FU: -43,742,637€	FU: -16,348€
		No	0.34	0.86	3,624 €				TS: -309€		SS: -35,948,071€	SS: -13,435€
	CG	Yes	0.63	0.91	4,009 €	0.88	3,996 €		HC: -334€		HC: -38,856,491€	HC: -14,522€
		No	0.37	0.82	3,975 €				AC: -340€		AC: -39,554,512€	AC: -14,783€
Knee	IG	Yes	0.47	0.89	5,050 €	0.86	4,907 €	0.025	FU: -376€	2,416	FU: -36,333,850€	FU: -15,040€
		No	0.53	0.84	4,781 €				TS: -309€		SS: -29,859,467€	SS: -12,360€
	CG	Yes	0.46	0.86	5,670 €	0.84	5,283 €		HC: -334€		HC: -32,275,282€	HC: -13,360€
		No	0.54	0.82	4,953 €				AC: -340€		AC: -32,855,077€	AC: -13,600€

Legend: MCID = minimal clinically important difference; p = transition probability; QALY = quality-adjusted life years; E(Q) = expected QALYs; E(C) = expected healthcare expenditure; ICER = incremental cost-effectiveness ratio; p.a. = per annum; p.p. = per patient; IG = intervention group; CG = control group; FU = follow-up expenditure only (no intervention cost); TS = Trial Setting; HC = Hybrid Care; AC = Automated Care.

Cost columns report 12-month follow-up healthcare expenditure only; TS/HC/AC add the scenario-specific intervention cost per patient. In this combined variation (licence €20; implementation €10), intervention cost per patient is: TS €67 = personnel €37 (62×€0.59) + implementation €10 + licence €20; HC €42 = €12 + €10 + €20; AC €36 = €6 + €10 + €20.

ICERs were calculated as $[E(C)_{IG} - E(C)_{CG}]$ + intervention cost per patient (TS/HC/AC) divided by ΔQALYs. Negative ICERs indicate dominance.

[1] Considering only 12-month follow-up costs without intervention costs

[2] Assuming 62% of 187,640 = 116,337 annual primary total hip and 62% of 155,859 = 96,633 total knee arthroplasties that are performed in the large hospital size category with >500 cases per year (following Endoprothesenregister Deutschland (EPRD) - Jahresbericht 2024)

10. Sensitivity Analysis (iv) of Costs, QALYs, and ICERs

Table S10. Sensitivity Analysis (iv): QALY variation (reduced health benefit assumption)

Indication	Δ QALYs	Δ Healthcare Expenditure Savings p.p.	QALYs gained p.a.[1]	Annual Healthcare Expenditure Savings[2]	ICER
Hip	0.023	FU: -376€	4,316	FU: -70,552,640€	FU: -16,347€
		TS: -218€		TS: -40,905,520€	TS: -9,478€
		HC: -243€		HC: -45,596,520€	HC: -10,565€
	0.012	AC: -249€	2,158	AC: -46,722,360€	AC: -21.652€
Knee	0.025	FU: -376€	3,896	FU: -58,602,984€	FU: -15,042€
		TS: -218€		TS: -33,977,262€	TS: -8,721€
		HC: -243€		HC: -37,873,737€	HC: -9,721€
	0.013	AC: -249€	1,948	AC: -38,808,891€	AC: -19.920€

Δ QALYs = per-patient QALY difference (intervention − control); Δ Healthcare Expenditure Savings p.p. = per-patient 12-month healthcare-expenditure difference (negative = savings); QALYs gained p.a. = national annual QALYs (hip and knee reported separately); Annual Healthcare Expenditure Savings p.a. = national annual 12-month healthcare-expenditure difference (negative = savings).

ICER = [per-patient follow-up expenditure difference + intervention cost per patient (TS/HC/AC; FU adds €0)] ÷ ΔQALYs.

FU = follow-up expenditure only (no intervention cost). TS = Trial Setting (€158 per patient = €37 personnel + €21 implementation + €100 licence). HC = Hybrid Care (€133 = €12 + €21 + €100). AC = Automated Care (€127 = €6 + €21 + €100).

Costs reflect the statutory health-insurance perspective over 12 months; no discounting.

[1]*Considering only 12-month follow-up costs without intervention costs as derived in the model of Figure 4*

[2]*Assuming 187,640 annual primary total hip and 155,859 total knee arthroplasties (following EPRD Annual Report 2024)*
